# A 3D Model of the Thermoelectric Microwave Power Sensor by MEMS Technology

**DOI:** 10.3390/s16060921

**Published:** 2016-06-21

**Authors:** Zhenxiang Yi, Xiaoping Liao

**Affiliations:** Key Laboratory of MEMS of the Ministry of Education, Southeast University, Nanjing 210096, China; xpliao@seu.edu.cn

**Keywords:** 3D model, power sensor, thermoelectric, GaAs MMIC, micro-electro-mechanical system (MEMS) technology

## Abstract

In this paper, a novel 3D model is proposed to describe the temperature distribution of the thermoelectric microwave power sensor. In this 3D model, the heat flux density decreases from the upper surface to the lower surface of the GaAs substrate while it was supposed to be a constant in the 2D model. The power sensor is fabricated by a GaAs monolithic microwave integrated circuit (MMIC) process and micro-electro-mechanical system (MEMS) technology. The microwave performance experiment shows that the S_11_ is less than −26 dB over the frequency band of 1–10 GHz. The power response experiment demonstrates that the output voltage increases from 0 mV to 27 mV, while the incident power varies from 1 mW to 100 mW. The measured sensitivity is about 0.27 mV/mW, and the calculated result from the 3D model is 0.28 mV/mW. The relative error has been reduced from 7.5% of the 2D model to 3.7% of the 3D model.

## 1. Introduction

Power sensors are always applied in microwave communication systems and wireless applications. Recently, Dehe *et al.* proposed a kind of thermoelectric power sensor based on the Seebeck effect [1,2,3]. This sensor excels in good performance, *i.e.,* in low return loss, high sensitivity, and good linearity. In order to understand the inherent mechanism, Kozlov presented the one-dimensional (1D) heat transfer model of this power sensor [4,5]. In our group, based on this work, Wang *et al.* established the 1D model of the thermoelectric power sensor fabricated by a GaAs MMIC process [6,7]. In the 1D model, all hot junctions in the thermopiles are assumed to be with same temperature. Obviously, it does not accord to the facts in application. Therefore, a two-dimensional (2D) model of the power sensor was established [8,9]. In this model, the temperature of the thermopile is different, determined by the distance to the heated resistors. Unfortunately, error still exists, and the calculated temperature from the 2D model is less than the FEM and the experiment result. The reason is that the heat flux density in the thickness direction of the substrate is considered to be a constant. Obviously, this assumption in the 2D model is not accurate, and it actually decreases from the upper surface to the lower surface. The reason is that the thermoelectric power sensor is fabricated on the substrate, and the load resistors produce heat at the upper surface. Therefore, the heat transfers in the substrate and the heat flux density decrease in the thickness direction of the substrate. Finally, in application, the inaccurate 2D model will have an effect on the temperature compensation and sensitivity improvement of the power sensor. Moreover, up to now, there is no literature on the three-dimensional (3D) model of the thermoelectric power sensor.

For these reasons, in order to reduce the error and obtain a more accurate result, a new 3D model of the thermoelectric power sensor is proposed in this paper.

## 2. Modeling

**Figure 1:**
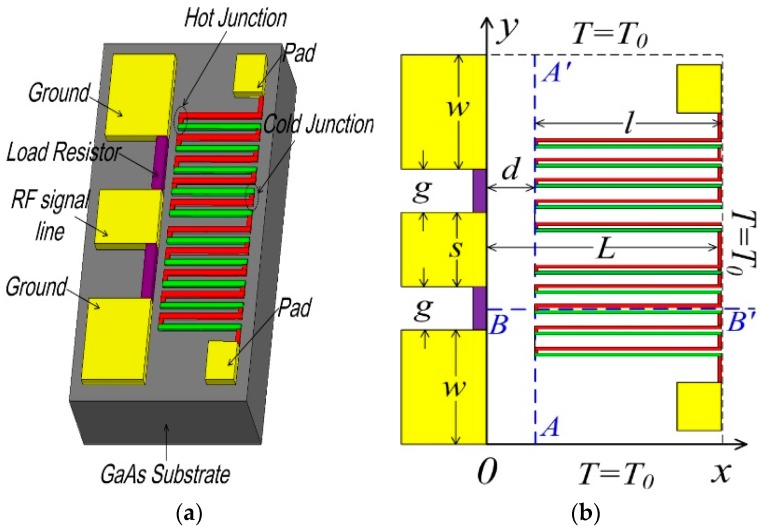
(**a**) Schematic overview and (**b**) dimension parameters of the thermoelectric microwave power sensor.

Figure 1a gives the schematic overview of the thermoelectric microwave power sensor by micro-electro-mechanical system (MEMS) technology. The microwave power is fed by a coplanar waveguide (CPW) transmission line and dissipated by two load resistors. The produced heat transfers in the substrate and is measured by DC voltage based on the Seebeck effect. Figure 1b shows the dimension parameters of the power sensor in the 3D model.

**Figure 2:**
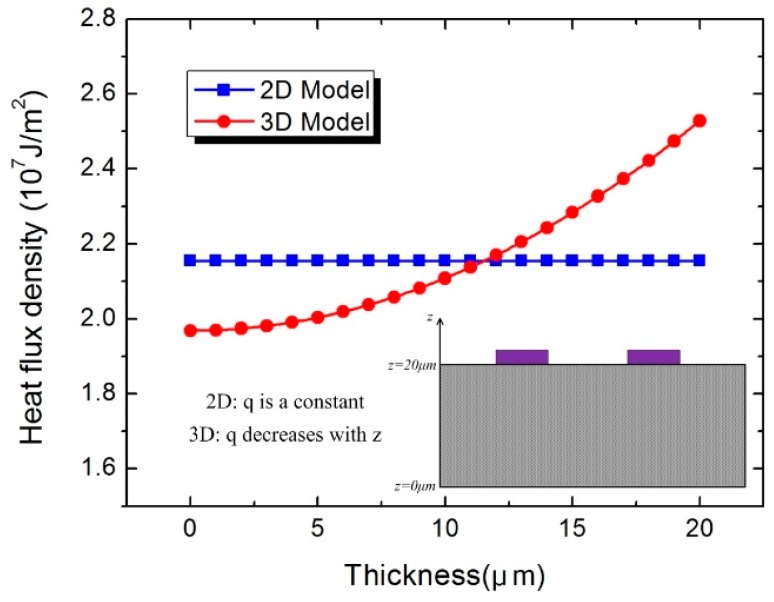
Heat flux density of the power sensor in the thickness direction of the substrate.

In practice, the heat flux density decreases in the thickness direction from the upper surface to the lower surface. In this paper, it is supposed to be proportional to the square of the thickness, as shown in Equation (1):

q = az^2^ + b (1)

where q is the heat flux density, z is the coordinate in the thickness direction of the substrate, and a and b are coefficient, respectively. In this model, a = 3.125 × 10^3^, and b = 2 × 10^7^. Figure 2 shows the heat flux density of the power sensor in the thickness direction of the substrate. As can be observed, the heat flux density decreases from 2.5 × 10^7^ J/m^2^ at the upper surface to 2.0 × 10^7^ J/m^2^ at the lower surface of the substrate.

Based on the heat transfer equation, the temperature distribution of the thermoelectric power sensor is a function of the *x* and *y*, and can be written as Equation (2) [8]:

(2) $$T=\sum_{n=1}^{+\infty }C_{n}(e^{\frac{n\pi }{W}x}-e^{\frac{n\pi }{W}(2L-x)})\sin\frac{n\pi }{W}y+T_{0},$$

where *C_n_* is the coefficients determined by the boundary consideration, *x* is the horizontal coordinate, *y* is the vertical coordinate, *W* is the width of the power sensor (*W* = 2w + 2g + s), and *T*_0_ is the ambient temperature.

**Figure 3:**
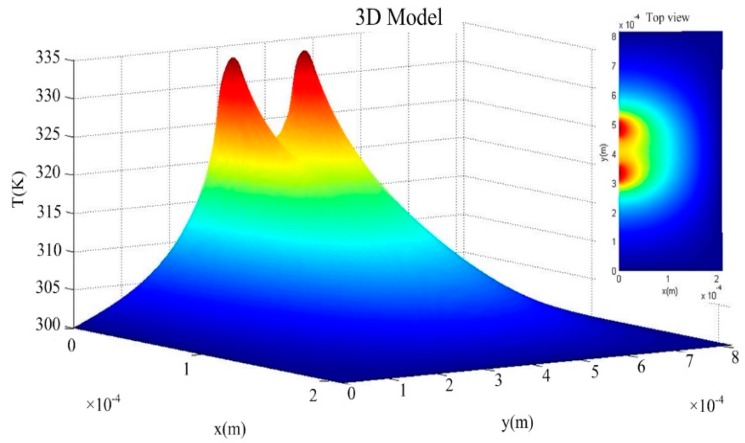
Calculated temperature distribution of the power sensor from the 3D model by the Matlab software.

Based on the parameters in Table 1, the Matlab software is used to draw the calculated temperature distribution of the power sensor from the 3D model under 100 mW. As shown in Figure 3, two peaks of the temperature are up to 335 K and appear in the center position of the load resistors.

## 3. Simulation

**Figure 4:**
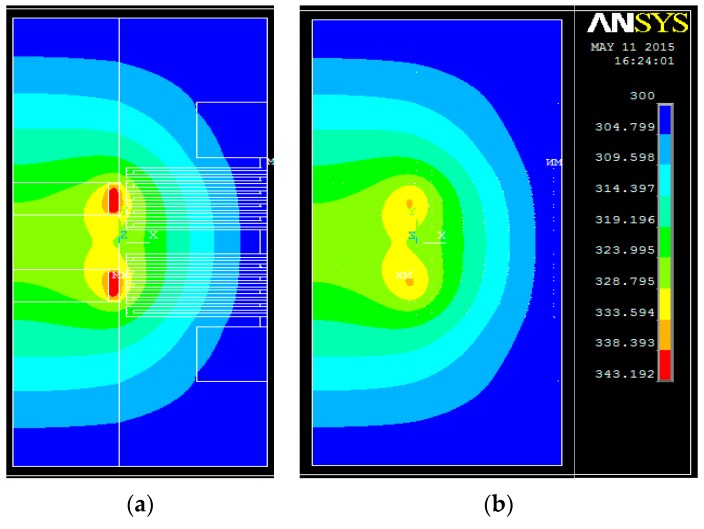
Simulated temperature distribution of the power sensor by the ANSYS software: (**a**) upper surface and (**b**) lower surface.

In order to verify the proposed 3D model, ANSYS software is applied to simulate the temperature distribution. The parameters of the power sensor equal to those in the model, as shown in Table 1. The mesh size of the substrate is 10 µm, the mesh size of the CPW transmission line and the load resistors are both 3 µm, and the mesh size of the thermopile is 3 µm. The ambient temperature is assumed to be 300 K and the boundary condition of the finite element method (FEM) model is that the temperature of the top face, bottom face, and right face equals to 300 K. The results are shown in Figure 4. Identical to the 3D model, the two load resistors have the highest temperature at 343 K. The produced heat transfers around in the substrate, and the temperature is mainly determined by the distance to the heated resistors.

**Figure 5:**
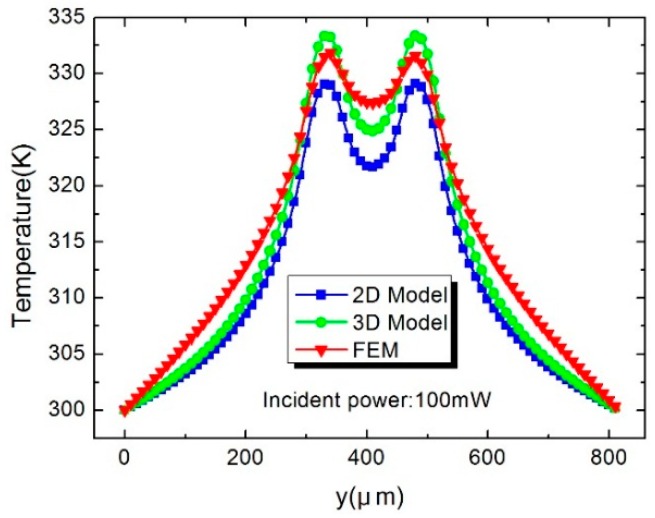
Temperature distribution in the AA′ (x = 10 µm) direction.

**Figure 6:**
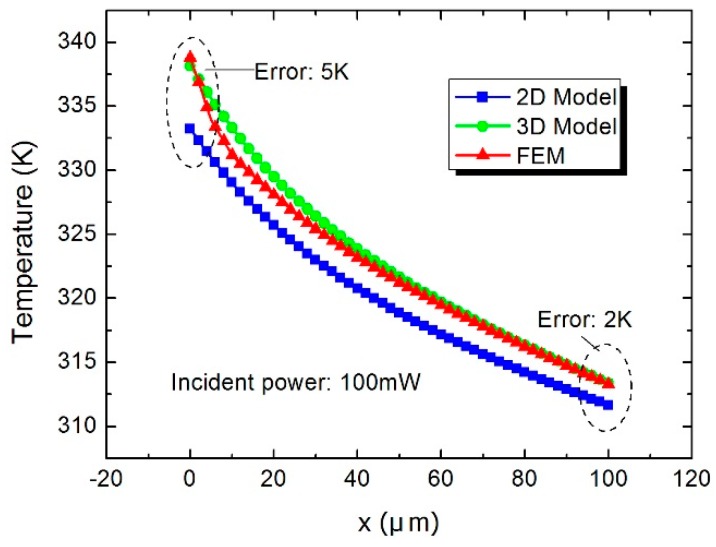
Temperature distribution in the BB′ (y = 279 µm) direction.

**Figure 7:**
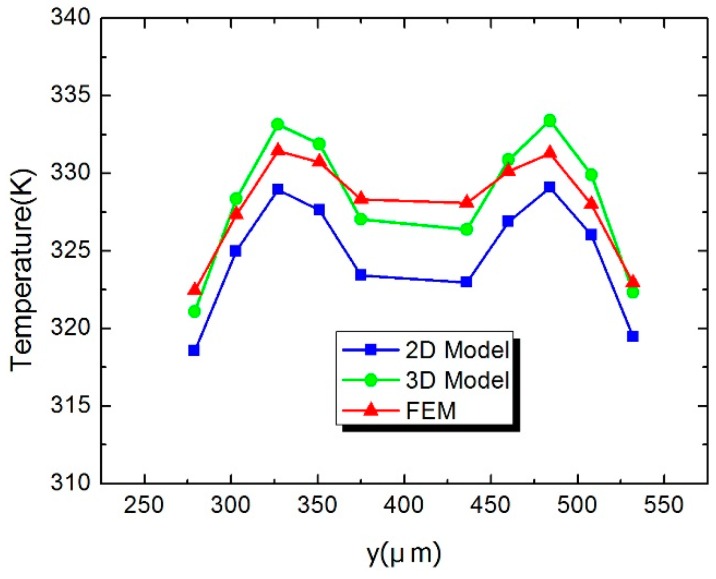
Temperature of hot junctions in ten thermopiles in the power sensor.

Figure 5 shows the temperature distribution (AA′ in Figure 1b) of the hot junctions in the thermopiles. Compared with the simulated results, the maximum temperature error of the 3D model is about 2.5 K, while it is up to 5.7 K in the 2D model. Figure 6 shows the temperature distribution (BB′ in Figure 1b) from the center of the load resistor to the cold junction of the thermopile. It demonstrates that temperature in the 3D model is higher than that in the 2D model, which is closer to the FEM results. The reason is that the heat flux density on the upper surface is higher than the average density in the 2D model. Figure 7 shows the temperature of the hot junctions of the ten thermocouples in the power sensors. As can be observed, the maximum error between the 3D model and the FEM results is about 2 K, while the 2D model is up to 5 K.

## 4. Experiment Evaluation

**Figure 8:**
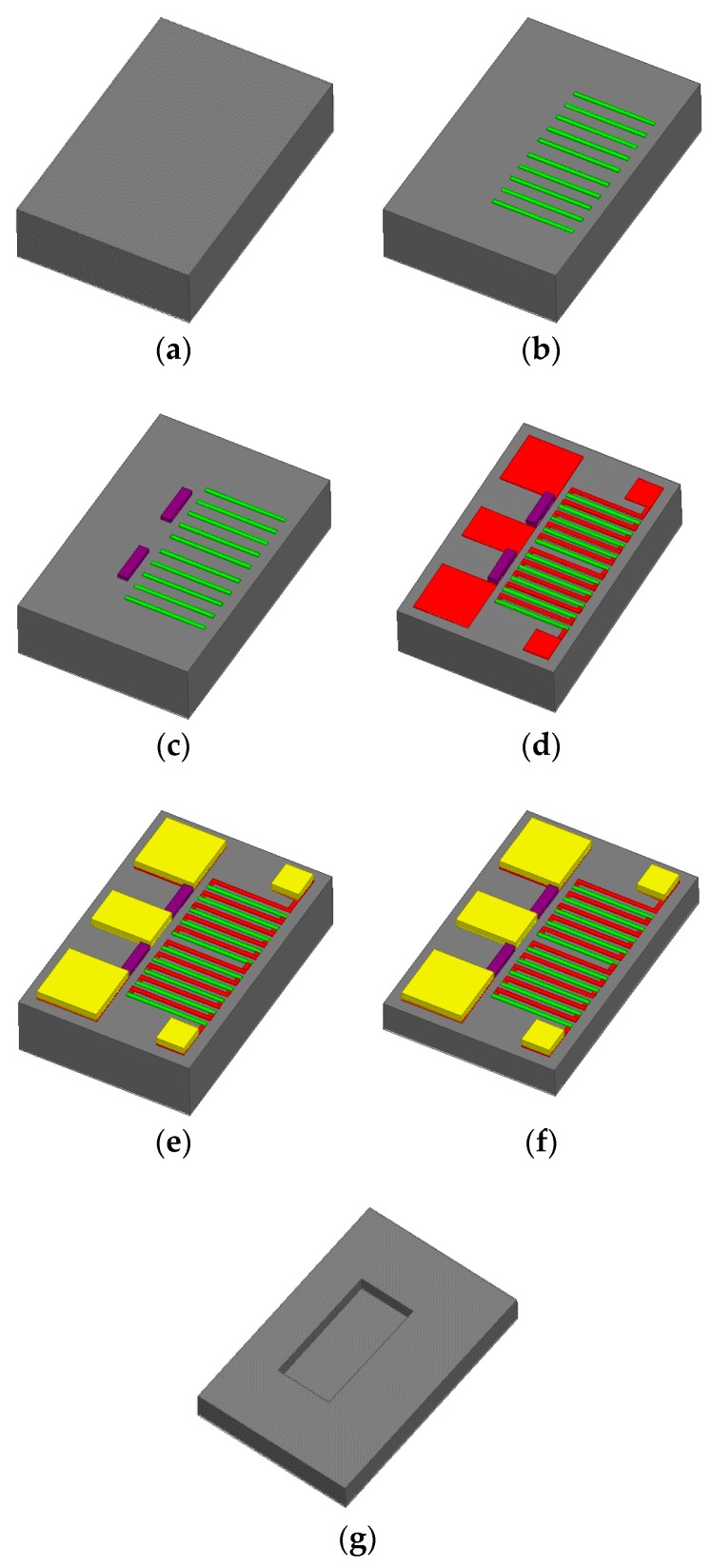
Fabrication process of the power sensor by GaAs MMIC technology (Steps (a)–(g) in Section 4).

The power sensor is fabricated using a GaAs MMIC process and MEMS technology. As shown in Figure 8, the fabrication steps are described as follows [10]:

(a) the GaAs substrate is prepared;
(b) the n^+^ GaAs thermopile is formed with a doping concentration of 1 × 10^18^ cm^−3^;
(c) a TaN layer is sputtered and patterned to form the load resistors and the square resistance is about 25 Ω;
(d) a 0.45-μm-thick Au layer is evaporated and patterned to form the CPW lines and contacting pads preliminarily;
(e) a 500/1500/300 A Ti/Au/Ti seed layer is evaporated and patterned, and, after removing the top Ti layer, a 2-μm-thick Au layer is electroplated to form the CPW transmission lines and the contacting pads;
(f) the GaAs substrate is thinned to 100 μm;
(g) in order to reduce thermal losses, the substrate underneath the thermopiles and the load resistors is etched to about 20 µm by MEMS technology.

**Figure 9:**
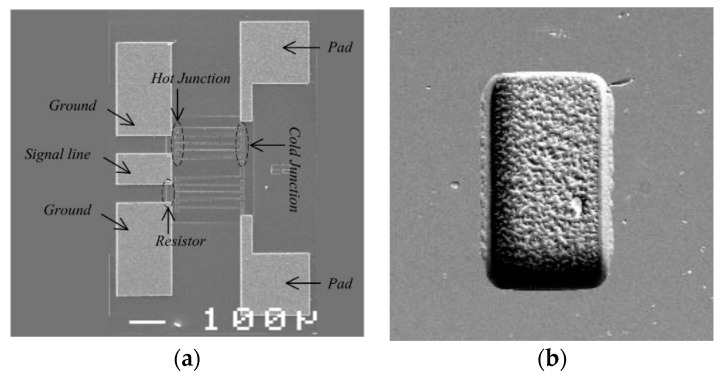
SEM photograph of (**a**) the power sensor and (**b**) the hole underneath the thermopiles.

Figure 8 gives the fabrication steps by GaAs MMIC process and MEMS technology. Figure 9 shows the SEM photograph of the fabricated power sensor and the hole underneath the thermopiles, respectively.

**Figure 10:**
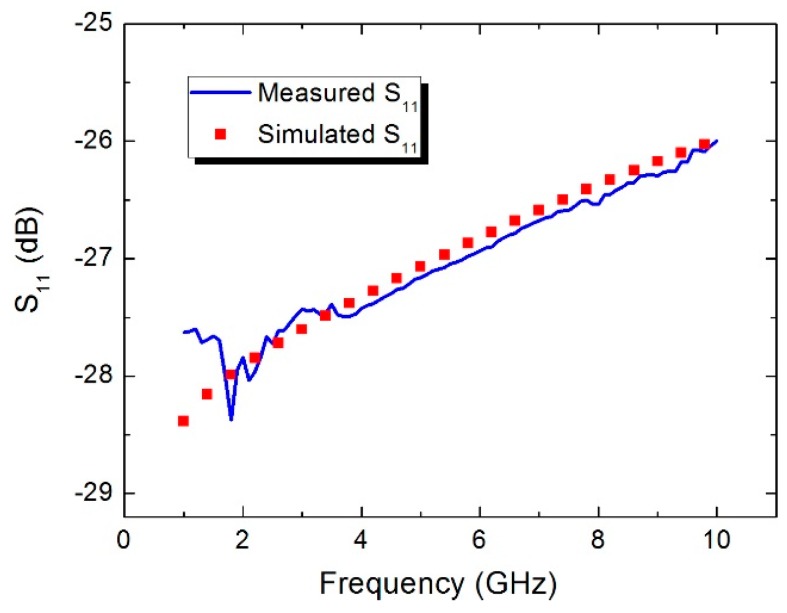
Measured S_11_ of the power sensor over the frequency band of 1–10 GHz.

The microwave performance of the fabricated sensor is tested by a network analyzer together with the microwave probing station. As shown in Figure 10, the measured S_11_ of the proposed power sensor is about −27.5 dB at 1 GHz, −27 dB at 5 GHz, and −26 dB at 10 GHz. The simulated result by High Frequency Structure Simulator (HFSS) software is also given in Figure 10. Clearly, a good agreement is obtained, demonstrating that the fabricated power sensor has good microwave performance over the frequency band of 1–10 GHz.

**Figure 11:**
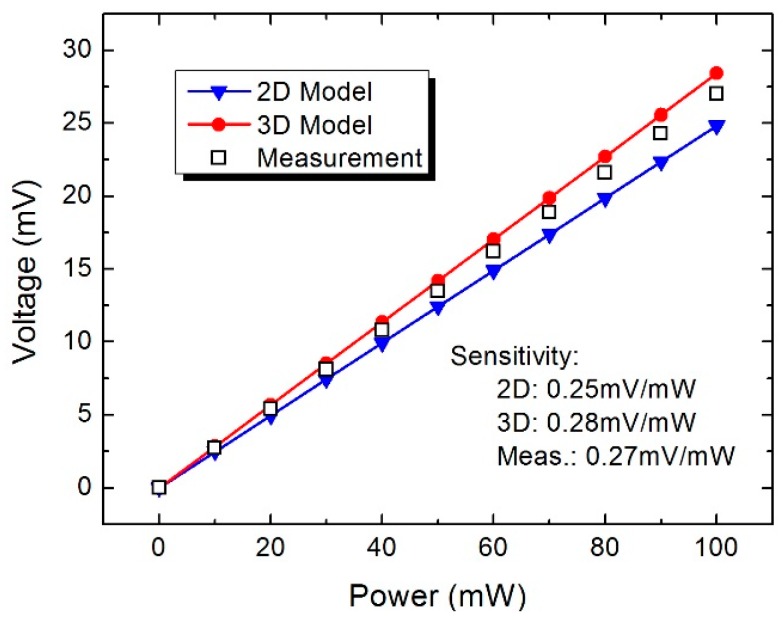
Measured voltage as a function of the incident power from 0 mW to 100 mW.

**Figure 12:**
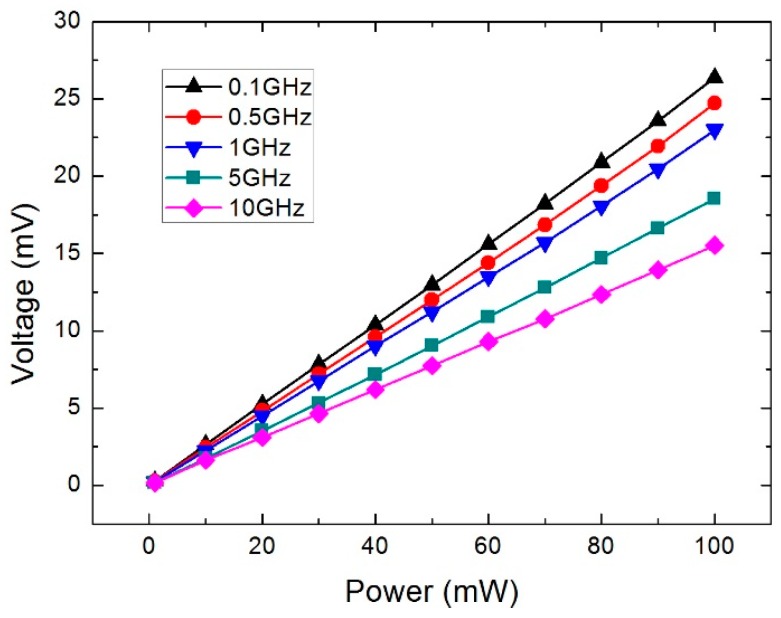
Measured voltage *versus* the incident power at 0.1 GHz, 0.5 GHz, 1 GHz, 5 GHz, and 10 GHz.

The microwave power measurement is performed using the analog signal generator, voltmeter, and microwave probing station. Figure 11 records the measured voltage as a function of the incident microwave power (DC power) from 1 mW to 100 mW. The output voltage increases from 0 mV to about 27 mV, and the sensitivity is close to 0.27 mV/mW. Meanwhile, the calculated results from the 2D and 3D model are also plotted in Figure 11. The sensitivity is close to 0.25 mV/mW and 0.28 mV/mW, respectively. As can be observed, the related error has been reduced from 7.5% of the 2D model to 3.7% of the 3D model. Figure 12 records the measured voltage as a function of the incident power at 0.1 GHz, 0.5 GHz, 1 GHz, 5 GHz, and 10 GHz, respectively. As can be observed, a linear relationship is obtained. Meanwhile, the sensitivity of the power sensor decreases with the frequency of the signal.

**Figure 13:**
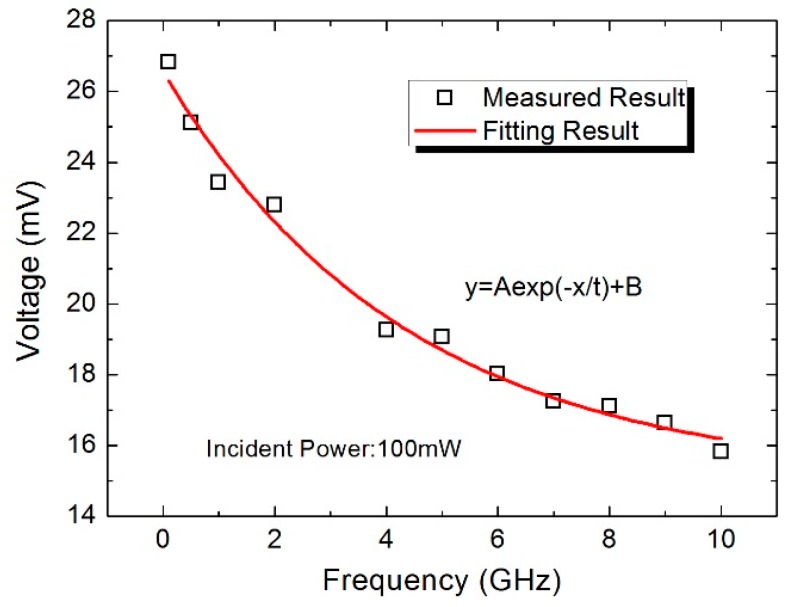
Measured voltage *versus* the frequency of the signal from 0.1 GHz to 10 GHz.

Figure 13 shows the measured voltage as a function of the frequency from 0.1 GHz to 10 GHz under the incident power of 100 mW. Clearly, the voltage decreases with the frequency of the signal, and a fitting curve is given. The voltage reduction in the power sensor is caused by a conductor loss and the dielectric loss of the transmission lines [11,12]. The loss from the signal generator to load resistors is caused by three parts: the coaxial line loss, the microwave probe loss, and the CPW transmission line loss. The coaxial line is about 1-m-long and plays an important role in the frequency loss. The high frequency will result in much loss in the transmission line. Therefore, the power reached to the load resistors decreases with the frequency. In addition, the parasitic loss of the load resistors also has an effect on the output DC voltage of the thermoelectric power sensor. The response time of this power sensor is close to 5 ms.

## 5. Conclusions

In summary, a new 3D model of the thermoelectric power sensor is presented to describe the temperature distribution in this paper. The Matlab software is applied to obtain the temperature and the ANSYS software is used to test the 3D model. The power sensor is fabricated by a GaAs MMIC process. The measured S_11_ is less than −26 dB from 1 GHz to 10 GHz, which agrees with the simulation in the HFSS software. The output voltage increases with the power linearly, and the sensitivity is close to 0.27 mV/mW. The experimental results demonstrate that related error has been reduced from 7.5% of the 2D model to 3.7% of the 3D model. This presented 3D model will be helpful for the sensitivity improvement and temperature compensation of power sensors in the future.

## Figures and Tables

**Table 1:** Parameters of the model.

Symbol	Quantity	Value
g	width of the CPW gap	58 μm
s	width of signal line	100 μm
w	width of CPW ground	300 μm
d	distance between the resistors and hot junctions in the *x* direction	10 μm
l	length of the thermocouples	200 μm
λ	thermal conductivity (GaAs)	46 W/(m·k)
a_1_	Seebeck coefficients (Au)	1.7 μV/K
a_2_	Seebeck coefficients (GaAs)	100 μV/K
T_0_	ambient temperature	300 K
a	coefficient 1	1.4 × 10^16^
b	coefficient 2	1.97 × 10^7^

## References

[1] Dehe A., Klingbeil H., Krozer V., Fricke K., Beilenhoff K., Hartnagel H.L. GaAs monolithic integrated microwave power sensor in coplanar waveguide technology. Proceedings of the IEEE MTT-S International Microwave Symposium Digest.

[2] Dehe A., Fricke-Neuderth K., Krozer V. Broadband thermoelectric microwave power sensors using GaAs foundry process. Proceedings of the IEEE MTT-S International Microwave Symposium Digest.

[3] Dehe A., Krozer V., Fricke K., Klingbeil H., Beilenhoff K., Hartnagel H.L. (1995). Integrated microwave power sensor. Electron. Lett..

[4] Kozlov A.G. (1999). Optimization of thin-film thermoelectric radiation sensor with comb thermoelectric transducer. Sens. Actuators A Phys..

[5] Kozlov A.G. (2000). Optimization of thin-film thermoelectric radiation sensor with separate disposition of absorbing layer and comb thermoelectric transducer. Sens. Actuators A Phys..

[6] Wang D., Liao X.P., Liu T. (2012). A thermoelectric power sensor and its package based on MEMS technology. J. Microelectromech. Syst..

[7] Wang D., Liao X.P., Liu T. (2012). Optimization of indirectly-heated type microwave power sensors based on GaAs micromachining. IEEE Sens. J..

[8] Yi Z.X., Liao X.P., Wu H. (2013). Modeling of the terminating-type power sensors fabricated by GaAs MMIC process. J. Micromech. Microeng..

[9] Yi Z.X., Liao X.P., Wu H. 2-D model of the indirectly-heated type microwave power sensor based on GaAs MMIC process. Proceedings of the IEEE Sensors.

[10] Zhang Z.Q., Liao X.P. (2012). GaAs MMIC fabrication for the RF MEMS power sensor with both detection and non-detection states. Sens. Actuators A Phys..

[11] Chramiec J., Adamski M.E., Kitlinski M. Evaluation of chip resistor CAD models used in microwave circuit design programs. Proceedings of the Signals Electronics Systems.

[12] Zhang Z.Q., Liao X.P. (2012). A three-channel thermoelectric RF MEMS power sensor for GaAs MMIC applications. Sens. Actuators A Phys..

